# ASCI: providing a forum for imaging scientists

**DOI:** 10.1186/s40679-018-0054-9

**Published:** 2018-03-10

**Authors:** Nigel D. Browning

**Affiliations:** School of Engineering, University of Liverpool, Liverpool, UK

Images are an essential part of the scientific discovery process in many research fields with the idiom “an image is worth a thousand words” holding true now more than ever. However, in many cases, these individual fields have developed their own methods for both acquiring and interpreting images based on new hardware and computational approaches, leading to a rapid divergence in what is considered state-of-the-art in imaging. This diversification means that in many cases, publications that could provide insights to imaging in general are published in field specific journals that are missed by the wider community. As the underlying mathematics for image interpretation is essentially the same, the goal of *Advanced Structural and Chemical Imaging* (ASCI) is to provide a forum that can unite scientists working in the imaging field and lead to a rapid transition of new approaches from a single demonstration case into a widely implemented method.

ASCI is published by SpringerOpen, a suite of fully open access journals that is part of the wider Springer Nature group. The journal enjoys a growing readership with an ever-increasing number of papers receiving double-digit citations. The metrics of the journal as it enters its fourth year are consistent with the leading imaging journals in electron microscopy and are trending upward. As the journal matures, it is worth reflecting on where we came from and where we will be heading in the future.

Images are used all the time by biologists, clinicians, physicists, chemists, materials scientists and engineers to provide unique insights into the structures and/or the dynamic processes they are working to understand and control. While each type of image generates contrast through a different bio-chemical–physical process, the interpretation of the contrast map, i.e., the image, for any application uses similar mathematical methods—all methods use a mathematical definition of spatial resolution, for example. The aim of ASCI is, therefore, to provide a widely accessible forum whereby experts in imaging in different fields can demonstrate how they apply both traditional and new mathematical methods to interpret contrast in their images. In addition, any modification they make to their instrumentation to take advantage of the mathematical methods can be shared with experts in other fields with the goal of disseminating and employing new technologies over a wider sphere of influence.

In discussing images, we have to remember that almost everything can be classified as an image—even the text in this article can be analyzed using mathematical methods for image analysis. For ASCI, we are open to the publication of any discussion of image acquisition and analysis, but we anticipate that the majority of papers will focus on the analysis of 1-D (spectra), 2-D (traditional images), 3-D (tomograms) and energy filtered (hyperspectral) images acquired through the selected detection of scattered light, X-rays, electrons, ions, neutrons and protons. Methods that can cross-cut multiple applications are particularly welcome in the journal.

A key focus of ASCI is on publishing special thematic article collections. These collections illustrate a particular imaging area that is contributing to the development of imaging technologies in general. While the thematic collections show the state-of-the-art in a given field, they also act as vehicles to disseminate the approaches to the wider imaging community. In addition, these thematic series are often linked to workshops and/or feature review articles that can also show the historical trend in how the field developed and where the disruptive insights came from. Such collections also serve to showcase the areas that the journal is interested in publishing, providing a well-established context for all groundbreaking new imaging research to be published. If you feel there is an important development in imaging (or a workshop being held in this area) that could benefit from a thematic article collection, the editorial team welcomes your suggestions and will work with you to provide the best open access resource for publication of the work.

The strength of ASCI versus other journals lies in the diversity of its audience with a specific expertise in the area of imaging. By bringing together imaging specialists, we can create a united platform that exhibits a range of research from across the imaging. ASCI will allow our authors and readers both to establish connections within the community and to provide an outlet for their research, which would be impossible through other publications. We invite everyone to join us on our process of accelerating scientific discovery through state-of-the-art imaging methods.

